# Effect of Salt Stress on the Activity, Expression, and Promoter Methylation of Succinate Dehydrogenase and Succinic Semialdehyde Dehydrogenase in Maize (*Zea mays* L.) Leaves

**DOI:** 10.3390/plants12010068

**Published:** 2022-12-23

**Authors:** Dmitry N. Fedorin, Alexander T. Eprintsev, Orlando J. Florez Caro, Abir U. Igamberdiev

**Affiliations:** 1 Department of Biochemistry and Cell Physiology, Voronezh State University, 394018 Voronezh, Russia; 2 Department of Biology, Memorial University of Newfoundland, St. John’s, NL A1C 5S7, Canada

**Keywords:** GABA shunt, NaCl, promoter methylation, salt stress, succinate dehydrogenase, succinic semialdehyde dehydrogenase, *Zea mays* L.

## Abstract

The effect of salt stress on the expression of genes, the methylation of their promoters, and the enzymatic activity of succinate dehydrogenase (SDH) and succinic semialdehyde dehydrogenase (SSADH) was investigated in maize (*Zea mays* L.). The incubation of maize seedlings in a 150 mM NaCl solution for 24 h led to a several-fold increase in the activity of SSADH that peaked at 6 h of NaCl treatment, which was preceded by an increase in the *Ssadh1* gene expression and a decrease in its promoter methylation observed at 3 h of salt stress. The increase in SDH activity and succinate oxidation by mitochondria was slower, developing by 24 h of NaCl treatment, which corresponded to the increase in expression of the genes *Sdh1-2* and *Sdh2-3* encoding SDH catalytic subunits and of the gene *Sdh3-1* encoding the anchoring SDH subunit. The increase in the *Sdh2-3* expression was accompanied by the decrease in promoter methylation. It is concluded that salt stress results in the rapid increase in succinate production via SSADH operating in the GABA shunt, which leads to the activation of SDH, the process partially regulated via epigenetic mechanisms. The role of succinate metabolism under the conditions of salt stress is discussed.

## 1. Introduction

Many areas on Earth are affected by salinity, and NaCl represents the most abundant salt causing salt stress in plants [1]. During the adaptation to salt stress conditions, plants involve the salinity tolerance mechanisms, which include limiting ion uptake, compartmentalizing ions, and preventing negative osmotic effects on cell functions. The capacity of mitochondrial respiration and its flexibility in the adaptation to changing environment are most essential for developing tolerance to salt stress [1]. This includes the support of efficient ATP generation, scavenging of reactive oxygen species (ROS), and the mitochondrial regulation of osmolyte concentrations. Succinate is accumulated in stress conditions in plants, and the increase in its oxidation in mitochondria represents one of important features of stress response [2]. This increase corresponds to the phenomenon of “salt respiration” [3], which is related to the modulation of redox balance in mitochondria and ROS formation [4], and the activation of respiratory enzymes and transporters at the transcriptional, translational, and post-translational levels [5]. 

The treatment with NaCl leads to the elevation of Ca^2+^, stimulates ROS formation and facilitates the transport of ions in plants [5]. These processes influence the tricarboxylic acid (TCA) cycle [6] and affect the exchange of tri- and dicarboxylic acids between mitochondria and cytosol [7]. This results in the increase in succinate and other TCA cycle intermediates [8,9], while the mitochondrial respiration shifts from the conversion of 2-oxoglutarate in the TCA cycle to the γ-aminobutyric acid (GABA) shunt [10]. In particular, GABA shunt affects the homeostasis of succinate and γ-hydroxybutyrate and controls ROS accumulation in the conditions of salinity, which was demonstrated in the studies of transgenic tomato plants with differential expression of three GABA shunt enzymes [11]. The GABA shunt represents an important source for succinate production in stress conditions, and the mechanisms of coordinated regulation of the GABA shunt and succinate dehydrogenase (SDH) are essential in the strategy of plant biochemical adaptation to stress [6,10].

Recently, it was shown that circular RNAs are involved in reprogramming the genomic function during adaptation to salt stress in maize [12]. It was also revealed that salt stress causes several epigenetic changes [13] including histone modifications [14], alterations of DNA methylation processes, and causes other epigenetic changes [15]. The degree of promoter methylation in salt stress conditions can be under control of brassinosteroids and other phytohormones [16], in particular, the genes affected by methylation include those that are involved in cell cycle, development, and several metabolic processes [17]. In the previous study, we demonstrated that epigenetic mechanisms are involved in triggering changes of respiratory metabolism in the conditions of salinity [18]. In particular, the mitochondrial forms of aconitase and fumarase are activated and the cytosolic forms of these enzymes are suppressed in the conditions of salt stress, and these changes are mediated by the epigenetic mechanisms of promoter methylation of their genes. 

In the present study, we investigated the effect of NaCl on the activity of the succinate-producing enzyme of the GABA shunt, succinic semialdehyde dehydrogenase (SSADH), and on the activity of SDH, as well on the rates of succinate oxidation in mitochondria of maize. This plant is the third most important food crop globally after wheat and rice, and the first most important C_4_ crop. Our aim was to explore the possibility of the coordinated regulation of the GABA shunt and the TCA cycle at the level of SSADH and SDH. We studied the expression and promoter methylation of the gene encoding SSADH (*Ssadh1*) and of the genes encoding four subunits of SDH: *Sdh1-2* encoding the flavoprotein subunit SDHA, *Sdh2-3* encoding the iron-sulfur subunit SDHB, *Sdh3-1* and *Sdh4* encoding the anchoring subunits SDHC and SDHD [19]. We demonstrate that salt stress results in the increase in SSADH expression and activity and in the increase in expression of the catalytic subunits and one anchoring subunit of SDH. It was shown that epigenetic mechanisms are involved in the expression of SSADH and of the iron-sulfur subunit of SDH. As a result, the capacity for succinate oxidation in mitochondria increases, which is accompanied by a higher involvement of the alternative pathway of mitochondrial respiration. 

## 2. Results

### 2.1. Effect of Salt Stress on SSADH Activity and Expression

**Figure 1:**
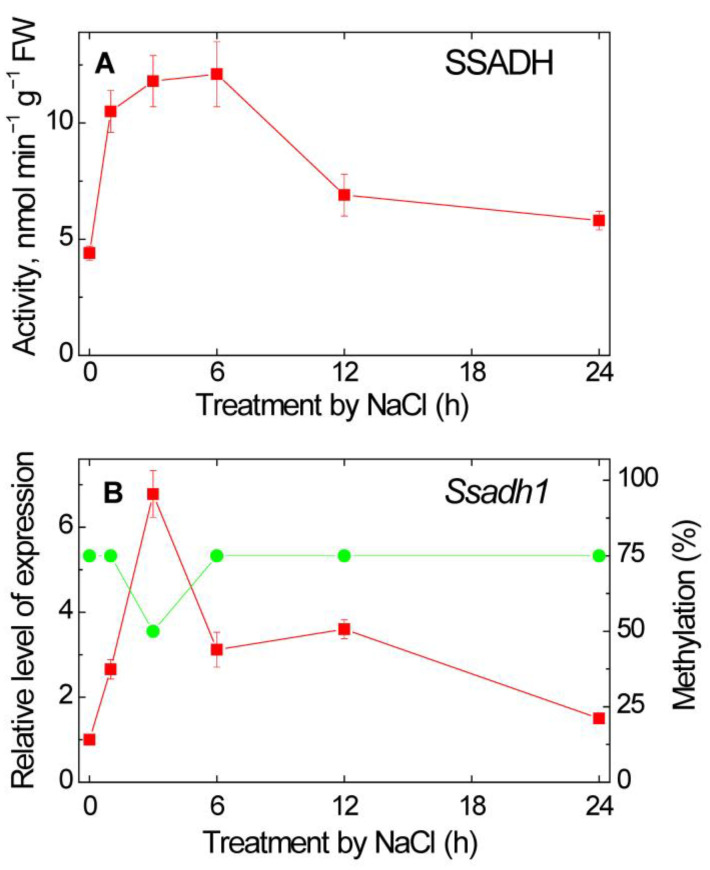
Succinic semialdehyde dehydrogenase (SSADH) activity (**A**), and expression (red squares) and promoter methylation (green circles) of the *Ssadh1* gene (**B**) after the transfer of maize seedlings in 150 mM NaCl. The data on activity and expression represent the means of three biological repeats ± SD. The control (untreated) did not exhibit statistically significant changes in the variation level.

The incubation of maize seedlings in 150 mM NaCl solution for 24 h led to a threefold increase in the activity of SSADH that peaked at 6 h of NaCl treatment, followed by a decline to a level which exceeded the initial level by ~1.5 times at 12–24 h of incubation (Figure 1A). The increase in SSADH activity was preceded by an elevation of the *Ssadh1* gene expression and a decrease in its promoter methylation (Figure 1B). The relative level of *Ssadh1* gene transcripts, determined using the developed primers for RT-PCR (Table S1), reached a maximum value by 3 h of salt stress, exceeding the control values by ~4.5 times, followed by a decrease in the mRNA levels. Using methyl-specific primers developed on the basis of the nucleotide sequence of the *Ssadh1* gene promoter (Table S2), it was shown that 3 h of salt stress led to a decrease in the methylation of the promoter from 75 to 50%, then the degree of methylation returned to 75% and remained at a constant level (Figure 1B).

### 2.2. Effect of Salt Stress on SDH Activity and on the Mitochondrial Succinate Oxidation

**Figure 2:**
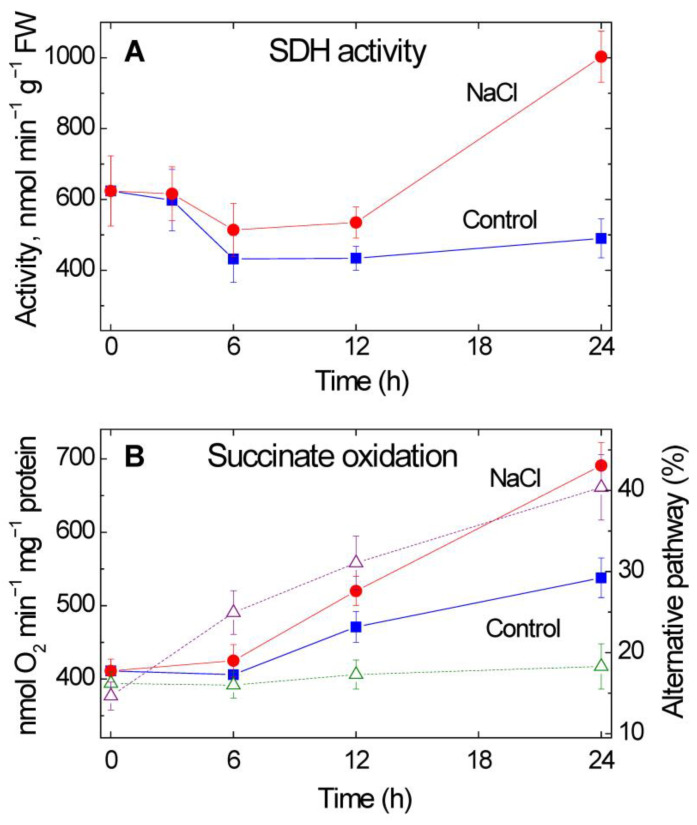
Succinate dehydrogenase (SDH) activity (**A**) and the rate of succinate oxidation by mitochondria isolated from maize leaves (**B**) after the transfer of maize seedlings in 150 mM NaCl. The triangles and dotted lines indicate the involvement of cyanide-resistant (alternative) pathway in succinate oxidation. The data represent the means of three biological repeats ± SD.

The increase in SDH activity was slower than the increase in SSADH activity, the activity becoming almost twice higher in the salt stressed plants by 24 h of NaCl treatment (Figure 2A), which corresponded to a 40% increase in the rate of succinate oxidation by isolated mitochondria (Figure 2B). This increase was accompanied by a stronger inhibition of succinate respiration by KCN, which demonstrates a higher involvement of the cyanide-resistant pathway in salt stressed plants after 24 h incubation of plants in 150 mM NaCl (Figure 2B). 

### 2.3. Effect of Salt Stress on the Expression of Succinate Dehydrogenase Genes and Methylation of Their Promoters

**Figure 3:**
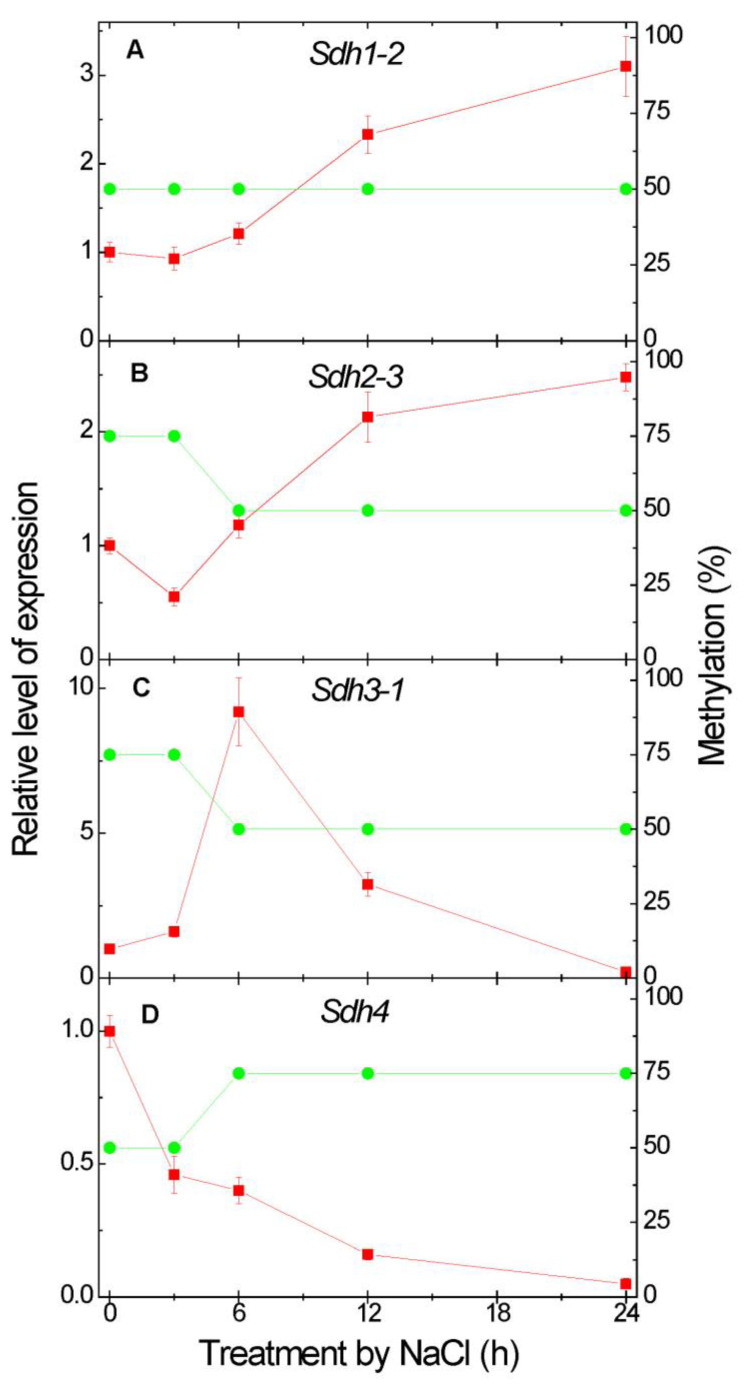
Expression (red squares) and promoter methylation (green circles) of the *Sdh1-2* (**A**), *Sdh2-3* (**B**), *Sdh3-1* (**C**) and *Sdh4* (**D**) genes after the transfer of maize seedlings in 150 mM NaCl. The expression data represent the means of three biological repeats ± SD. The control (untreated) did not exhibit statistically significant changes in the variation level.

The exposure to NaCl induced a 2–3-fold increase in the expression of the genes *Sdh1-2* and *Sdh2-3* encoding correspondingly the catalytic flavoprotein and the iron-sulfur protein subunits, which was observed in 12–24 h (Figure 3A,B). The expression of the genes *Sdh3-1* and *Sdh4* encoding the anchoring subunits did not correlate with the activity of SDH decreasing significantly by 24 h of NaCl treatment (Figure 3C,D). Salt stress inhibited the expression of the *Sdh4* gene throughout the experiment, while the expression of the *Sdh3-1* gene strongly increased in the first 6 h of exposure to NaCl and then declined. The primers developed to study the expression of SDH subunits are presented in Table S1. 

Using the methyl-specific primers developed on the basis of the nucleotide sequence of the promoters of the four investigated SDH genes (Table S3), it has been established that the *Sdh1-2* gene is characterized by a stable methyl status of the studied CG dinucleotides under the salt stress at the level of 50% (Figure 3A). The increase in the *Sdh2-3* expression was accompanied by the decrease in promoter methylation. In the first hours of NaCl exposure, the *Sdh2-3* gene promoter was methylated at the level of 75%, and then, starting from 6 h, the methylation status of the CG dinucleotides of the *Sdh2-3* gene promoter decreased to 50% and remained at this level up to 24 h of plant exposure to 150 mM NaCl (Figure 3B).

Expression of the gene *Sdh3-1* in the first 3 h of salt stress corresponded to the degree of methylation of the studied CG dinucleotides at the level of 75%, and later, starting from 6 h the degree of methylation decreased and remained at the level of 50% (Figure 3C). For the gene *Sdh4*, the increase in promoter methylation was observed by 6 h from 50 to 75% and remained at this level until the end of the experiment (Figure 3D). 

## 3. Discussion

The obtained data demonstrate that salt stress leads to a rapid increase in succinate production via SSADH operating in the GABA shunt (Figure 1), which then results in the activation of SDH (Figure 2). This activation is achieved by the increase in expression of SSADH (Figure 1) and of the catalytic subunits (SDHA and SDHB) and one anchoring subunit (SDHC) of SDH (Figure 3), which is regulated via the epigenetic mechanism of promoter methylation of the genes encoding SSADH and the SDH subunits SDHB and SDHC. The activity of the GABA shunt in plants increases rapidly in response to various biotic and abiotic stresses [11,20]. This is associated with the physiological role of the GABA shunt in preventing the decrease in pH under stress conditions [21,22] and in reducing the formation of ROS [11,23]. Due to the ability of SSADH to reduce NAD^+^ and to form succinate under stress conditions, when TCA is inhibited and ROS increases, the rate of ROS accumulation is controlled [23]. The accumulation of GABA caused by biotic or abiotic stressors (hypoxia, salinity, cold, dehydration) and its conversion via the GABA shunt can prevent ROS accumulation and further cell death in stress conditions [23,24,25]. The GABA shunt is also essential for plants to grow normally and be able to withstand stress due to the need to maintain a certain level of several metabolites [22]. 

In the conditions of TCA cycle inhibition that occur in stress conditions, in particular, upon the elevation of redox level [26], the GABA shunt operates as an important bypass that supports the TCA cycle operation [23,27,28]. In this modified TCA cycle, SSADH performs the reduction of NAD^+^ and the formation of succinate. The obtained data show that the increase in SSADH activity is provided by the increase in transcription of the *Ssadh1* gene via changing the methyl status of its promoter (Figure 1). The rapid activation of SSADH in maize leaves in the conditions of salinity (Figure 1) reorganizes the TCA cycle to bypass the mitochondrial enzymes that are inhibited under the salt stress [2]. This maintains a high level of synthesis of energy equivalents due to the additional supply of the respiratory substrate to the TCA cycle. For this, the GABA shunt and SSADH as its component are activated, which provides the necessary redox status in cells under salt stress [10]. The observed phenomenon of the GABA shunt activation represents the strategy when the alternative metabolic pathways and shunts overcome the salinity-induced inhibition of central carbon metabolism [2]. 

The increase in succinate formation via the GABA shunt can explain the observed increase in the activity of SDH and correspondingly in the rate of succinate oxidation by mitochondria (Figure 2A,B). The induction of SDH in conditions of salt stress was previously observed in *Amaranthus caudatus* L. [29] and strawberry (*Fragaria vesca* L.) [30]. This increase follows the marked increase in the expression of the genes *Sdh1-2* and *Sdh2-3* encoding correspondingly the catalytic flavoprotein subunit SDHA and the iron-sulfur protein subunit SDHB (Figure 3A,B). In the case of the increase in the *Sdh2-3* expression, the epigenetic mechanism of regulation of promoter methylation is involved, while for the *Sdh1-2* gene the level of promoter methylation does not change. The expression of the genes *Sdh3-1* and *Sdh4* encoding correspondingly the anchoring subunits SDHC and SDHD do not correlate with the activity of SDH decreasing significantly by 24 h of the NaCl treatment, which suggests that primarily the regulation of catalytic subunits is essential for the promotion of the observed changes in SDH activity and succinate oxidation. An increase in expression of the gene *Sdh3-1* after 6 h incubation of plants in 150 mM NaCl indicates the possible involvement of the subunit SDHC encoded by this gene in the regulation of SDH activity, with the involvement of the epigenetic control in this regulation. The choice of particular genes encoding SDH subunits is connected with their high transcript abundance in maize seedlings, which was determined in the previous studies [31,32,33]. 

**Figure 4:**
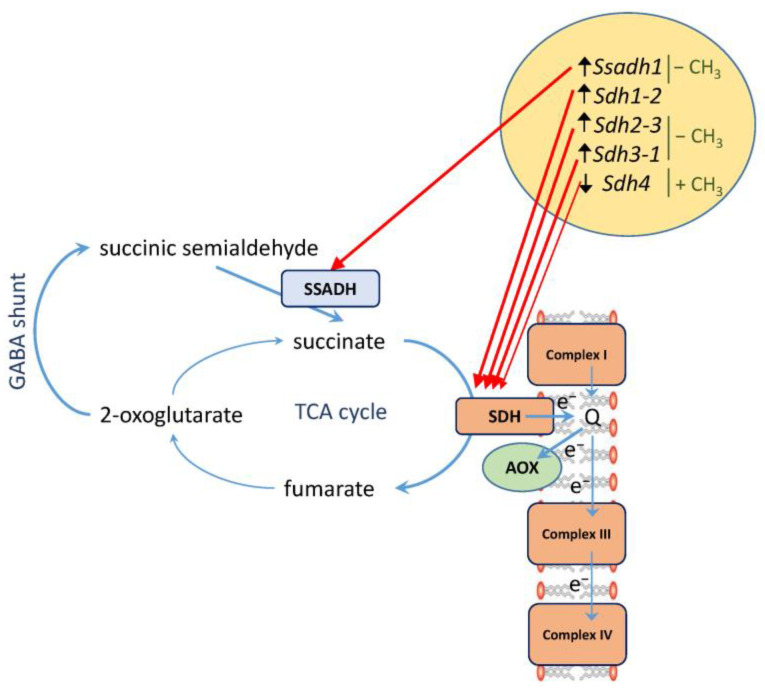
Scheme of succinate metabolism in maize leaves under salt stress. In the conditions of salt stress, the TCA cycle is bypassed via the GABA shunt. Succinic semialdehyde dehydrogenase (SSADH) supplies succinate to succinate dehydrogenase (SDH) which three subunits are induced at the level of transcription of the corresponding genes, while the expression of the fourth subunit is suppressed. The changes in expression are controlled by methylation of promoters of the corresponding genes (− CH_3_, decrease in methylation level; + CH_3_, increase in methylation level). The flow of electrons via alternative oxidase (AOX) is increased. Thick arrows indicate the processes that are activated, thin arrows show the processes that do not change or are suppressed.

The increase in the rate of succinate oxidation is accompanied by the twofold increase in the engagement of the cyanide-resistant pathway capacity (Figure 2B). This indicates that stress conditions lead to the induction of the mitochondrial alternative oxidase insensitive to cyanide, which supports a higher capacity for the oxidation of succinate and other TCA cycle substrates. This mechanism plays an important role in cell bioenergetics in stress conditions (Figure 4). The important role of alternative oxidase in the maintenance of metabolism and in prevention of ROS formation in the conditions of salt stress has been demonstrated in several studies [34,35,36,37,38]. The involvement of alternative oxidase in “salt respiration” can explain the observed lower rate of nitric oxide (NO) production in salt stressed plants [39], which differs from the hypoxic stress when NO production increases [40]. 

Previously we showed that the change in promoter methylation level of the genes encoding SDH subunits represents the mechanism of its regulation in the conditions of hypoxia [19] and in the course of germination [32]. While under hypoxia this leads to the inhibition of SDH and concomitant accumulation of succinate, salt stress, on the contrary, results in the activation of SDH, which is mediated by the increase in expression of the two catalytic subunits. These studies demonstrate that methylation of promoters is a common mechanism of the regulation of SDH activity in the conditions of various stresses. 

The results obtained in this study demonstrate that in the leaves of maize plants subjected to salt stress, the rapid increase in succinate production via the GABA shunt takes place. This is correlated with the activation of succinate oxidation in mitochondria via the cyanide insensitive pathway. The epigenetic mechanism of promoter methylation is involved in providing the increase in expression of SSADH and of the iron-sulfur SDH subunit, while the increase in expression of the flavoprotein SDH subunit is not related to the epigenetic mechanism. Succinate production in the GABA shunt and its oxidation in the conditions of salt stress represent an important mechanism of reorganization of cell bioenergetics to provide rapid adaptation to the changing environment. 

## 4. Materials and Methods

### 4.1. Object of Investigation

The two-week-old seedlings of maize (*Zea mays* L., cv Voronezhskaya 76, obtained from the Voronezh branch of the All-Russian Research Institute of Maize) were used in this study. The plants were grown hydroponically without the addition of mineral nutrients at 12 h of daylight with intensity of 90 µmol quanta m^−2^ s^−1^ at an ambient temperature of 25 °C. These conditions were optimal for a short-day C_4_ plant of tropical origin. The effect of salt stress was studied by placing the plants with a previously removed root system in a 150 mM aqueous solution of NaCl for 24 h, the plants placed in water for 24 h were used as a control. The concentration was sufficient for inducing changes in the metabolism without severe damage of the plants [41]. The samples were taken after 1, 3, 6, 12 and 24 h from the start of the experiment to explore a short-term response of plants to salt stress.

### 4.2. Determination of Succinic Semialdehyde Dehydrogenase and Succinate Dehydrogenase Activities

For the measurement of enzymatic activities, which was performed using the spectrophotometer SF-2000 (OKB Spectr, St. Petersburg, Russia), maize leaves were homogenized at 4 °C in 50 mM Tris-HCl buffer, pH 7.5, containing 1 mM EDTA, 10 mM KCl and 1 mM MgCl_2_ and centrifuged at 10,000*× g* for 10 min. Succinic semialdehyde dehydrogenase (SSADH; EC 1.2.1.24) activity was measured by change in optical density at 340 nm caused by the reduction of NAD^+^ during the oxidation of succinic semialdehyde [42]. Succinate dehydrogenase (SDH; EC 1.3.5.1) was measured at 600 nm using the artificial electron acceptor dichlorophenolindophenol (DCPIP) in the medium containing 30 mM potassium phosphate buffer, pH 7.8, 1 mM phenasine methosulfate (PMS), 0.08 mM DCPIP, 2 mM sodium azide and 20 mM sodium succinate [43]. All chemicals were obtained from Sigma-Aldrich (St. Louis, MO, USA). The unit of enzymatic activity corresponds to the formation of 1 nmol product per minute at 25 °C. 

### 4.3. Succinate Oxidation by Isolated Mitochondria

Mitochondria were isolated at 4 °C from leaves using the extraction buffer containing 50 mM HEPES (pH 7.2), 210 mM mannitol, 70 mM sucrose, 1 mM EGTA, and 0.5% (*w*/*v*) fatty acid-free bovine serum albumin (BSA). The homogenate was filtered through five layers of cheesecloth. The filtrate was centrifuged at 1500*× g* for 5 min at 4 °C. The resulting supernatant was centrifuged at 10,000*× g* for 15 min at 4 °C. The precipitate was washed with extraction buffer and centrifuged again at 10,000*× g* for 15 min at 4 °C. The resulting mitochondrial pellet was resuspended in the extraction buffer and used to measure oxygen uptake.

Oxygen uptake was measured at 25 °C and standard atmospheric pressure (101.32 kPa) using a Clark type electrode and an Oxytherm system (Hansatech Instruments Ltd., Pentney, UK), in a chamber with 1 mL of respiratory buffer containing 10 mM Tris-HCl (pH 7.2), 225 mM mannitol, 75 mM sucrose, 10 mM KCl, 5 mM KH_2_PO_4_ and 0.1% BSA (*w*/*v*). The suspension of mitochondria (50 µL) isolated at 0, 6, 12 and 24 h incubation of the plants on 150 mM NaCl and from the control plants were added in the chamber flowed by the addition of 5 mM succinate. After the stabilization of O_2_ uptake (State 2), ADP (final concentration 100 nM) was added resulting in the State 3 O_2_ uptake until ADP was depleted (State 4). The inhibition of cytochrome *c* oxidase was achieved by adding 1 mM KCN. Cyanide-resistant mitochondrial respiration was determined as the rate of oxygen uptake after the addition of KCN as inhibitor, assuming 100% as the rate of oxygen consumption by mitochondria in the oxidative phosphorylation state before the addition of the inhibitor [44,45]. The rate of succinate oxidation was expressed in nmol O_2_ min^−1^ mg^−1^ protein.

### 4.4. RNA Isolation and PCR Analysis

Total cellular RNA was isolated from the leaves of the studied plants by phenol-chloroform extraction using LiCl as a precipitant [46]. The selection of primers was performed on the basis of nucleotide sequences presented in the international GenBank database using the Primer-Blast program. The reverse transcription reaction was performed using the MMLV reverse transcriptase kit (Evrogen ZAO, Moscow, Russia) according to the manufacturer’s recommendations. Real-time PCR was performed on a LightCycler 96 instrument (Roche, Solna, Sweden) using Taq polymerase (Evrogen ZAO, Moscow, Russia) according to the manufacturer’s instructions. SYBR Green I (Sigma-Aldrich, St. Louis, MO, USA) was used as an intercalating dye. The primers are presented in the Supplementary Table S1. Amplification was carried out according to the following parameters: preliminary denaturation, 95 °C, 5 min, followed by 35 cycles, each including the stages 95 °C—10 s; 56–59 °C—10 s; 72 °C—10 s. At the end, a 10 min final elongation was performed at 72 °C. Quantitative control of the matrix was performed using amplification of the Ef-1α elongation factor gene [47]. The values of the relative levels of transcripts of the studied genes were calculated using the 2^−ΔΔ*C*^_T_ method [48].

For the selection of primers for methylation-specific PCR (MS-PCR) the program MethPrimer—Li Lab, UCSF was used [49,50], see Supplementary Tables S2 and S3 for the full set of methyl-specific primers. The quantitation of MS-PCR was performed on the basis of the results of electrophoregrams of PCR products. The values of the degree of promoter methylation are the cumulative results of PCR analysis of the studied CG-dinucleotides in the promoter of each specific gene. Since there could be three types of result (non-methylated, partially methylated and fully methylated), the numerical calculation was performed in the following way. The value 0% was assigned when three studied CG-dinucleotides are non-methylated; 25%—one or two CG-dinucleotides are partially methylated; 50%—one or two CG-dinucleotides are methylated; 75%—one or two CG-dinucleotides are partially methylated, and the remainder are fully methylated; 100%—all three CG dinucleotides are methylated [32].

### 4.5. Statistical Analysis

The experiments were performed in three biological and four analytical replicates. Data were subjected to two-way analysis of variance (ANOVA) using STATISTICA version 9 data analysis software (Statsoft Wipro, East Brunswick, NJ, USA). Results are presented as average means and standard deviations. Statistically significant differences at *p* < 0.05 are discussed.

## 5. Conclusions

Salt stress results in the rapid increase in succinate production via the GABA shunt leading to the activation of succinate oxidation in mitochondria. The increase in the expression of SSADH and of the iron-sulfur SDH subunit is regulated via the epigenetic mechanism of promoter methylation of their genes. Succinate formation in the GABA shunt followed by an increase in its mitochondrial oxidation represents an important mechanism of the rapid metabolic adaptation of maize plants to the conditions of salt stress. 

## Data Availability

All of the data are already provided in the main manuscript. Contact the corresponding author if further information or explanation is required.

## Supplementary Materials

The following supporting information can be downloaded at: https://www.mdpi.com/article/10.3390/plants12010068/s1, Table S1: Nucleotide sequences of primers to the genes encoding succinic semialdehyde dehydrogenase and the subunits of succinate dehydrogenase. Table S2: Primers to the promoter of the succinic semialdehyde dehydrogenase gene for methyl-specific PCR. Table S3: Primers to the promoter of the succinate dehydrogenase gene for methyl-specific PCR.

## Abbreviations

GABA	γ-aminobutyric acid
ROS	reactive oxygen species
SDH	succinate dehydrogenase
SSADH	succinic semialdehyde dehydrogenase
TCA	tricarboxylic acid
