# Supplementary material for: Effect of Salt Stress on the Activity, Expression, and Promoter Methylation of Succinate Dehydrogenase and Succinic Semialdehyde Dehydrogenase in Maize (Zea mays L.) Leaves
Source: Plants (Basel). 2022 Dec 23;12(1):68. doi: 10.3390/plants12010068 (PMC9823291; doi:10.3390/plants12010068)
Supplement: Supplementary file 1 [file plants-12-00068-s001.zip › plants-2116576-supplementary.pdf]

Table S1. Nucleotide sequences of primers to the genes encoding succinate-semialdehyde dehydrogenase and the subunits of succinate dehydrogenase.

Gene	Primer	Nucleotide sequence	T* (°C)
Ssadh-1	Forward	CACAGCCTGGGGATGTCATT	58
	Reverse	GGTGCCACCTGCGTTTGTAT	
Sdh1-2	Forward	CAAACGGGTCACTTCCAACT	56
	Reverse	CCAAAACTGTCCCACGTCTT	
Sdh2-3	Forward	GAGAGGCTACAGGCAATAACTGAG	58
	Reverse	AGTTTTAAACATTGATTCTTATTG	
Sdh3	Forward	AAGGAGGCTTCTCCATCTCC	59
	Reverse	CAGAGCTGCTACAGGGGAAG	
Sdh4	Forward	AACCCACTGGGAAGAGACCT	59
	Reverse	GACGATTGACGCGAGAATTT	

*Annealing temperature

Table S2. Primers to the promoter of the succinate-semialdehyde dehydrogenase gene for methyl-specific PCR

Gene	Position of the studied cytosine		Primer*	Nucleotide sequence
Ssadh-1	I	−615	Forward M	GTATTGTTGAGTGTCGTGTGG
			Reverse M	TCTTACTATATTAAAACAACTCCATTTCCAC
			Forward U	GTATTGTTGAGTGTTGTGTGG
			Reverse U	TCTTACTATATTAAAACAACTCCATTTCCAC
	II	−720	Forward M	GAGTGAAGATGAATATACGATGAATTT
			Reverse M	TCTTACTATATTAAAACAACTCCATTTCCAC
			Forward U	GAGTGAAGATGAATATATGATGAATTT
			Reverse U	TCTTACTATATTAAAACAACTCCATTTCCAC
	III	−215	Forward M	GACGGATTTATGAAGAGTTTAAGGG
			Reverse M	TCTTACTATATTAAAACAACTCCATTTCCAC
			Forward U	GATGGATTTATGAAGAGTTTAAGGG
			Reverse U	TCTTACTATATTAAAACAACTCCATTTCCAC

*The annealing temperature was 53–55 ^{o}C for all reactions

Table S3. Primers to the promoter of the succinate dehydrogenase gene for methyl-specific PCR

Gene		Position of the studied cytosine	Primer	Nucleotide sequence
Sdh1-2	I	−57	Forward M	AATATATTTTAAATTGCA
			Reverse M	TAATTTAAGGTTAGAGG
			Forward U	AATATATTTTAAATTACA
			Reverse U	TAATTTAAGGTTAGAGG
	II	−115	Forward M	TAACCCACCTTACAAGCA
			Reverse M	TAATTTAAGGTTAGAGG
			Forward U	TAACCCACCTTACAAACA
			Reverse U	TAATTTAAGGTTAGAGG
	III	−307	Forward M	CAAAATAAAACTATTGCT
			Reverse M	TAATTTAAGGTTAGAGG
			Forward U	CAAAATAAAACTATTACT
			Reverse U	TAATTTAAGGTTAGAGG
Sdh2-3	I	−109	Forward M	TTTTATACGATCGAGTTAGTACG
			Reverse M	AAAATATCTTTAAATAAATCTTAAACC
			Forward U	TTTTTTTATATGATTGAGTTAGTATG
			Reverse U	AAAATATCTTTAAATAAATCTTAAACC
	II	−165	Forward M	TTAATAATATCAACAAGCG
			Reverse M	TTAGTTATAAATTTTGATTG
			Forward U	TTAATAATATCAACAAACG
			Reverse U	TTAGTTATAAATTTTGATTG
	III	−205	Forward M	AAATCTTTCTTTCACCCGCT
			Reverse M	TTAGTTATAAATTTTGATTG
			Forward U	AAATCTTTCTTTCACCCACT
			Reverse U	TTAGTTATAAATTTTGATTG
Sdh3	I	−506	Forward M	TATTTTTATGTTTTTTATTTTGCGG
			Reverse M	TCAAGGGAACGAGTATATCTAAAC
			Forward U	TATTTTTATGTTTTTTATTTTGTGG
			Reverse U	TTAAGGGAATGAGTATATTTAAAT
	II	−454	Forward M	TATAATAAGGGTAAGTGAAAGTCGT

			Reverse M	TCAAGGGAACGAGTATATCTAAAC
			Forward U	TATAATAAGGGTAAGTGAAAGTTGT
			Reverse U	TTAAGGGAATGAGTATATTTAAAT
	III	−431	Forward M	TTTAGAGGGGAGGTGAATAGGCGA
			Reverse M	TCAAGGGAACGAGTATATCTAAAC
			Forward U	TTTAGAGGGGAGGTGAATAGGTGA
			Reverse U	TTAAGGGAATGAGTATATTTAAAT
Sdh4	I	−57	Forward M	GATGTTTTTTCGTCGTATTATTTTC
			Reverse M	GTATTAGGCGGTTTTAGAGAAGG
			Forward U	TGTTTTTTTGTTGTATTATTTTTGA
			Reverse U	GTATTAGGCGGTTTTAGAGAAGG
	II	−115	Forward M	TTTTAAAGTTTTTATTTTTTTCGA
			Reverse M	GTATTAGGCGGTTTTAGAGAAGG
			Forward U	TTTTAAAGTTTTTATTTTTTTTGA
			Reverse U	GTATTAGGCGGTTTTAGAGAAGG
	III	−307	Forward M	AAATTAGATTTAATTAATTTCGT
			Reverse M	GTATTAGGCGGTTTTAGAGAAGG
			Forward U	AAATTAGATTTAATTAATTTTGT
			Reverse U	GTATTAGGCGGTTTTAGAGAAGG
